# Moss and Liverwort Covers Structure Soil Bacterial and Fungal Communities Differently in the Icelandic Highlands

**DOI:** 10.1007/s00248-023-02194-x

**Published:** 2023-02-18

**Authors:** Javier Ortiz-Rivero, Isaac Garrido-Benavent, Starri Heiðmarsson, Asunción de los Ríos

**Affiliations:** 1 Department of Biogeochemistry and Microbial Ecology, National Museum of Natural Sciences (MNCN-CSIC), C/ Serrano 115 dpdo, E-28045 Madrid, Spain; 2 Departament de Botànica i Geologia, Fac. CC. Biològiques, Universitat de València, C/ Doctor Moliner 50, E-46100 Burjassot, Valencia Spain; 3 Icelandic Institute of Natural History, Akureyri Division, Borgir Nordurslod, 600 Akureyri, Iceland; 4 Present address: Northwest Iceland Nature Research Centre, Aðalgötu 2, 550 Sauðárkrókur, Iceland

**Keywords:** Cryptogamic cover, Bryophyte, Iceland, Metabarcoding, Microbial ecology, Polar areas

## Abstract

Cryptogamic covers extend over vast polar tundra regions and their main components, e.g., bryophytes and lichens, are frequently the first visible colonizers of deglaciated areas. To understand their role in polar soil development, we analyzed how cryptogamic covers dominated by different bryophyte lineages (mosses and liverworts) influence the diversity and composition of edaphic bacterial and fungal communities as well as the abiotic attributes of underlying soils in the southern part of the Highlands of Iceland. For comparison, the same traits were examined in soils devoid of bryophyte covers. We measured an increase in soil C, N, and organic matter contents coupled with a lower pH in association with bryophyte cover establishment. However, liverwort covers showed noticeably higher C and N contents than moss covers. Significant changes in diversity and composition of bacterial and fungal communities were revealed between (a) bare and bryophyte-covered soils, (b) bryophyte covers and the underlying soils, and (c) moss and liverworts covers. These differences were more obvious for fungi than bacteria, and involved different lineages of saprotrophic and symbiotic fungi, which suggests a certain specificity of microbial taxa to particular bryophyte groups. In addition, differences observed in the spatial structure of the two bryophyte covers may be also responsible for the detected differences in microbial community diversity and composition. Altogether, our findings indicate that soil microbial communities and abiotic attributes are ultimately affected by the composition of the most conspicuous elements of cryptogamic covers in polar regions, which is of great value to predict the biotic responses of these ecosystems to future climate change.

**Supplementary Information:**

The online version contains supplementary material available at 10.1007/s00248-023-02194-x.

## Introduction

Bacteria and fungi are major biotic components of edaphic ecosystems globally, where they bring invaluable ecosystem services thanks to their role in organic matter degradation, hence driving biogeochemical cycles, and also by their contribution to soil formation and stabilization [1–6]. Their ability to form mutualistic relationships with roots becomes pivotal for subsequent colonization of recently deglaciated areas by higher plants [3, 7, 8]. In fact, these microorganisms are the dominant components of soil biomass in ice-free areas of polar regions, including permafrost [9–11]. The soil surface of these regions is frequently covered by highly structured communities encompassing cyanobacteria, lichenized and non-lichenized fungi, as well as bryophytes, which are collectively referred as to cryptogams, i.e., plant or plant-like organisms that reproduce by spores instead of seeds [12, 13]. These multi-organism cryptogamic covers are essential for nutrient cycling dynamics, especially in areas where vascular plants are less dominant, as they fuel food webs with the products of photosynthesis and nitrogen fixation [12, 14–16]. For example, in the Arctic tundra, species forming cryptogamic covers are the main primary producers, together with small shrubs, grasses, and non-graminoid herbs [17], whereas in Antarctic polar deserts, they have been considered as the most important sources of carbon and nitrogen [18]. Furthermore, the establishment of cryptogamic covers in these regions determines not only the soil abiotic attributes, but also the dynamics of co-occurring edaphic microbial communities [16].

Bryophytes, including mosses (phylum *Bryophyta*) and liverworts (phylum *Marchantiophyta*), are among the most conspicuous, photosynthetic components of cryptogamic covers occurring in the polar tundra [19, 20], with a considerable contribution to soil formation and stabilization through the deep penetration of rhizoids and protonemata, which also accelerate physical and chemical weathering processes [15, 21, 22]. In Iceland, they often extend over large expanses of the island [23, 24]. These non-vascular plants often tolerate desiccation and wide temperature fluctuations [25], thanks to their poikilohydric nature [1]. In spite of the importance of these peculiar covers for primary productivity at polar regions, and especially for Icelandic terrestrial ecosystems, the effects that their establishment exert on soil development and their contribution to edaphic microbial diversity have been rarely studied [26–28], and often only from the bacterial perspective [29, 30].

The main aim of the present study was to determine whether cryptogamic covers dominated by different bryophyte lineages (mosses and liverworts) exert similar effects on the edaphic abiotic and biotic attributes. To this end, the diversity and structure of bacterial and fungal communities associated with the establishment of both types of cryptogamic covers around Mt. Hekla in the southern part of the Icelandic Highlands was analyzed by DNA metabarcoding and the spatial structure of these covers characterized by scanning electron microscopy in backscattered electron mode (SEM-BSE). The potential roles of fungi occurring in moss and liverwort-dominated covers were also examined and compared. Therefore, the findings of this work provide insight into the inner workings of polar tundra ecosystems.

## Material and Methods

### Study Area and Experimental Design

**Fig. 1:**
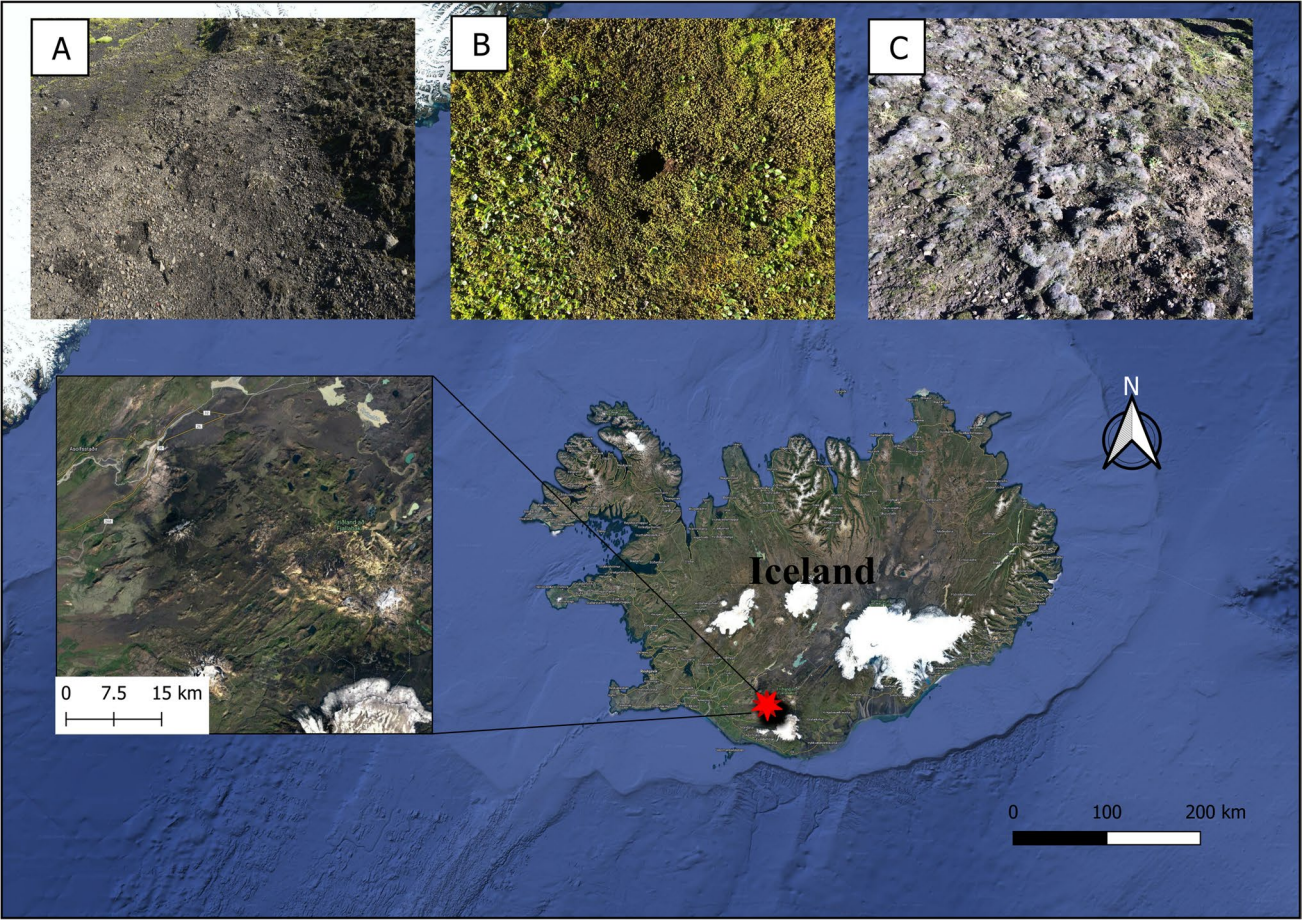
Map of Iceland indicating the placement of the Highlands. Overview of the different types of soil where the collection of the samples took place: (**A**) bare soil; (**B**) soil dominated by mosses; and (**C**) soil dominated by liverworts. Photos: A. de los Rios

Soils with and without cryptogamic covers dominated by mosses or liverworts were collected in July 2017 in a flat area at Suðurland region (63°55′59″ N, 20°59′49″ W; 625 m), near to the Hekla volcano (Fig. 1), in the Highlands region of Iceland. The sampling area shows a sub-arctic climate, with an average annual temperature of 3 °C, and annual rainfall ranging from 600 to 1500 mm, most of it as snow [31, 32]. Soils are of the Andosol type, mostly with a volcanic origin [33]. The dominating mosses in the sampling site were *Niphotrichum ericoides* (Brid.) Bednarek-Ochyra & Ochyra and a *Pohlia* sp., and the prevailing liverwort was *Anthelia juratzkana* (Limpr.) Trevis., which extended over large areas [25, 34, 35]. The taxonomic identity of mosses was confirmed by a molecular phylogenetic analysis (data not shown).

Fifteen samples of the upper soil layer were randomly collected at a minimum distance of 1 m between them using a 5-cm diameter stainless steel corer. Five corresponded to soils with cryptogamic covers dominated by mosses, another five dominated by liverworts and the remaining 5 without bryophyte cover (Fig. 1). A profile of 0–5-cm deep of bare soil was taken, whereas profiles of approximately 7-cm deep were collected at areas with bryophyte cover. In the last ones, the uppermost 2-cm band corresponding to the cryptogamic cover itself (henceforth referred as cover) was separated from the 5-cm band of underlying soil (henceforth referred as “soil below covers”) and stored independently. Soil samples were sieved (2-mm mesh) and thoroughly mixed to create one homogeneous composite sample per plot which was immediately preserved in RNAlaterTM (Thermo Fisher Scientific) until further processing. Cryptogam samples were frozen until processing.

### Analysis of Abiotic Soil Attributes

Carbon (C) and nitrogen (N) content and C/N ratio were determined in all the samples, i.e., soils below cryptogamic covers and bare soils, as well as the two bryophyte cover types. Organic matter content and pH were measured only in soil samples. Soil pH was determined with a Crison MicropH 2001 pH-meter in a soil-to-water ratio of 1:2.5 (mass/volume). Soil organic matter content was estimated by loss on ignition at 450 °C for 4 h [36]. Carbon and nitrogen content was calculated by dry combustion with an elemental analyzer (LECO TruSpec CN) at the CEBAS Ionomic Service (CSIC, Murcia).

Descriptive statistics were calculated for each soil attribute and results were graphically represented through bar diagrams constructed using the R package *ggplot2* [37]. ANOVA (or Kruskal-Wallis) tests were conducted to explore differences in soil variables among the various sampled soils and bryophyte covers. A *post hoc* test of multiple comparisons was performed with a Bonferroni correction. These analyses were done in RStudio version 4.0.4 [38].

### Laboratory Processing and High-Throughput Sequencing

Genomic DNA extraction from soil and cover samples was performed using the PowerSoil DNA Isolation Kit (MOBIO Laboratories, Carlsbad, CA, USA) following the manufacturer’s protocol. DNA concentration and quality were measured using a NanoDrop ND 1000 spectrophotometer (Thermo Fisher Scientific™). Fungal and bacterial DNA amplification followed the protocols suggested by the Earth Microbiome Project (available at http://www.earthmicrobiome.org/protocols-and-standards/16s/). For fungi, PCRs were carried out using the primer pair ITS1F_KYO2–ITS2_KYO2 [39] that targets the ITS1 region of the nuclear ribosomal internal transcribed spacer (nrITS). For bacteria, PCRs were carried out using the universal primer pair 515F–806R [40], which spans the V4 hypervariable region of the bacterial 16S rRNA genes. Fungal and bacterial amplicon libraries were then generated at the ASU Genomics Core (Arizona State University) and paired-end sequencing was performed on an Illumina MiSeq sequencer (version 2 module, 2 × 250), following the manufacturer’s instructions. Raw reads were demultiplexed and barcode sequences were removed by the sequencing center. The data sets generated for this study can be found in the NCBI Sequence Read Archive with the BioProject number PRJNA917534.

### Analysis of Bacterial 16S rRNA and Fungal ITS1 Data

Illumina data for fungi and bacteria were analyzed using the Divisive Amplicon Denoising Algorithm (DADA2) [41], which infers Amplicon Sequence Variants (henceforth referred as to ASVs) that differ from each other at least by a single nucleotide. Abundance tables containing inferred variants and read counts per samples for bacteria and fungi were obtained using R scripts available in the Microbiome Helper virtual box [42] (available at https://github.com/mlangill/microbiome_helper/wiki). The fungal dataset was also submitted to an ITSx extraction step [43] using a script made available by J.L. Darcy at GitHub (available at https://github.com/darcyj). These tables were subsequently converted into BIOM format, and then were further processed to remove all those ASVs that represented <0.005% of the total read abundance on a per-sample basis [41, 44], and to assign taxonomy metadata based on the UNITE [45] (version 8.2, available at https://unite.ut.ee/) and SILVA [46] (version 138, available at https://www.arb-silva.de/) databases for fungi and bacteria, respectively.

### Microbial Diversity Analyses

Taxonomic profiles of microbial communities at the phylum (bacteria) and class (fungi) levels were generated using default settings in Microbiome Analyst [47]. The numbers of ASVs that were shared among sample categories (soils and covers; bare soil and soil under cryptogamic covers), as well as those that were exclusive of any of the previous categories were graphically represented with Venn diagrams using *jvenn* [48]. Alpha diversity statistics (e.g., ASV richness, Shannon and Simpson indices, and Pielou’s evenness) were calculated using the R package *phyloseq* [49] based on community matrices with a rarefied read depth to 3925 and 25,276 which corresponded to the minimum library size obtained for fungi and bacteria, respectively (Fig. S1). The rarefaction of the data matrix does not affect the relative proportions of the read assignments, as these remain fairly constant regardless of the minimum read size [50]. Pielou’s evenness was calculated using the following formula: Shannon index/ln(richness). The function *Kruskal.test* was used to test for significant differences in relative abundances of fungi and bacteria among the different sample categories (covers and soils). Graphs were constructed using the *ggplot2* and *ggpubr* [51] R packages. The latter package allows adding the significance level obtained in each index.

Beta diversity analyses used the fungal and bacterial BIOM tables normalized by the cumulative sum scaling (CSS) method, which corrects for differences in sequencing depth between samples [52]. Non-metric multidimensional scaling (NMDS) was computed on the basis of Bray-Curtis dissimilarities to illustrate differences in the composition of microbial communities across sample categories. Ordination graphs were built with the *phyloseq* R package. To generate statistical support for any observed differences, the Analysis Of SIMilarities (ANOSIM) [53] as well as the non-parametric Adonis test [54] with 999 permutations were run in the R package *vegan* [55]. All tests regarded *p* values below 0.05 as significant.

Finally, the relationships between soil attributes and the fungal and bacterial community structures were examined and graphically represented using a distance-based redundancy analysis [56] (db-RDA) with the package *vegan*, following the scripts used by Garrido-Benavent et al. [57].

### Functional Properties of Fungal Communities

The database FungalTraits [58] was used to assign the potential role of fungi in the studied cryptogamic covers according to the different inferred ASVs. Life strategies were assigned for each ASV at the taxonomic level of genus. Two separate datasets were built: one considered the whole cryptogamic cover community, i.e., all sample categories jointly, and the other considered the five sample categories separately.

### Scanning Electron Microscopy Analysis of Cryptogamic Cover Structure

Small fragments of covers dominated by liverworts and mosses were processed for scanning electron microscopy in backscattered electron mode (SEM-BSE), following the methodology described in Wierzchos and Ascaso [59]. The samples were fixed in glutaraldehyde (3% v/v) and osmium tetroxide solutions (1% w/v), dehydrated in a graded ethanol series (from 30 to 100% v/v) and embedded in LR White resin. Blocks of resin-embedded rock-colonized samples were finely polished, carbon-coated, and observed using a FEI INSPECT microscope.

## Results

### Soil Attributes and Edaphic Microbial Communities

**Fig. 2:**
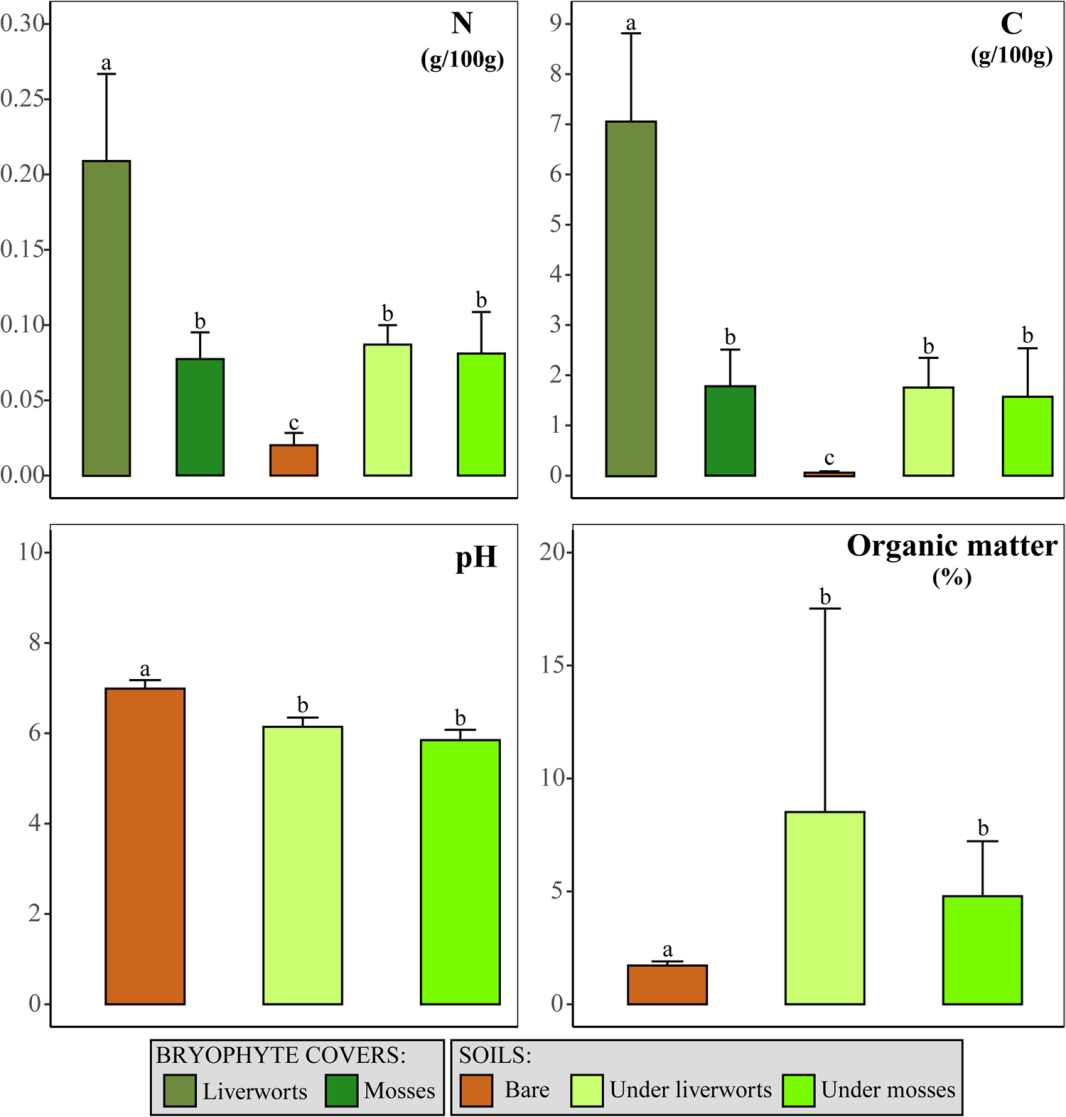
Bar diagrams (means ± standard errors) of soil attributes in the different types of soil and cryptogamic cover. Letters above bars indicate significant differences among categories (*p* value < 0.05)

The variables C, N, and organic matter content and the C/N ratio showed higher values in soils under bryophyte covers than in bare soils, whereas the reverse was true for pH (Fig. 2; Table S1). Liverwort covers showed significantly higher C and N contents than moss covers. All these differences were statistically supported (Table S2).

### Assessment of the Fungal Diversity, Abundance, and Specificity

**Fig. 3:**
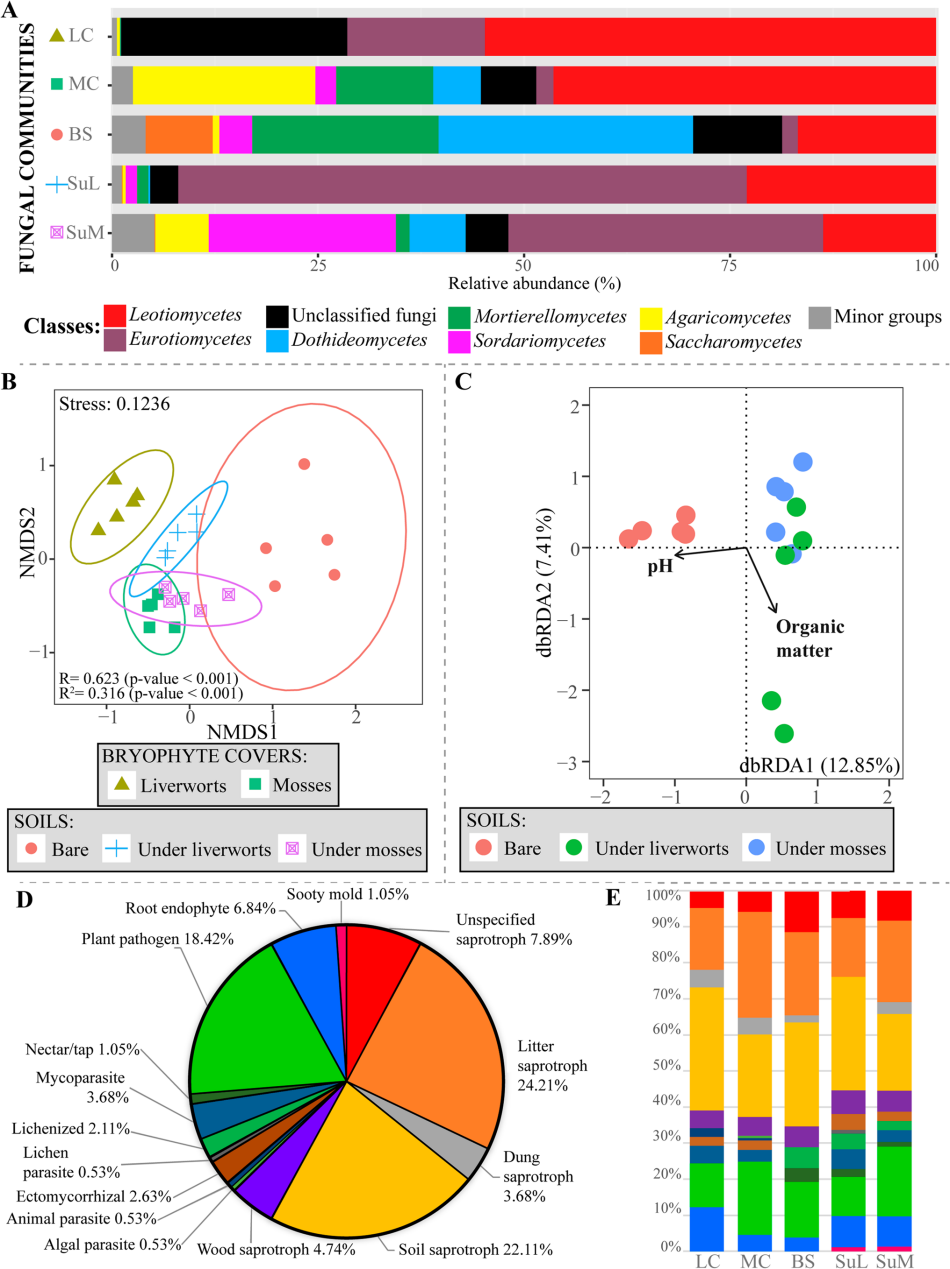
Overview of the fungal community structure and diversity revealed by high-throughput Illumina sequencing. **A** Relative abundances at the class level in the five studied communities (LC, liverwort cover; MC, moss cover; BS, bare soil; SuL, soil under liverworts; SuM, soil under mosses); “unclassified fungi” refer to the ASVs that could not been classified below the rank of kingdom, whereas “minor groups” include fungal classes below a certain value of relative abundance; **B** non-metric multidimensional scaling (NMDS) ordination plot of Bray-Curtis dissimilarities across sample categories, with ellipses representing the 95% confidence interval for a multivariate distribution and the results of ANOSIM and Adonis tests; **C** distance based redundancy analysis (db-RDA) with selected edaphic variables that explained most of the variability in the three soil fungal communities; **D** main ecological role of fungal ASVs considered at the genus level in the set of all samples and studied communities, or **E** in each community

The number of fungal ASVs that were inferred based on the 25 analyzed samples was 400. Rarefaction curves indicated that sequencing depth was sufficient to identify the majority of fungal ASVs in soils under liverworts and mosses, but probably insufficient for the other three studied communities (Fig. S1). The *Ascomycota* was the phylum showing the highest relative abundance (74%) in the studied communities, followed by *Mortierellomycota* (8%) and *Basidiomycota* (7%). *Rozellomycota*, *Monoblepharomycota*, and *Chytridiomycota* had lower relative abundances (data not shown). The ascomycete classes *Leotiomycetes* (38%) and *Eurotiomycetes* (20%) were the most abundant, followed by the *Dothideomycetes* (7%) and *Sordariomycetes* (5%), and classes *Mortierellomycetes* (8%) and *Agaricomycetes* (5%) in *Mortierellomycota* and *Basidiomycota*, respectively (Fig. 3A; Table S3). The orders *Helotiales* (31%), *Eurotiales* (11%), *Chaetothyriales* (9%), and *Mortierellales* (8%) (Fig. S2; Table S4), and the genera *Penicillium* (11%), *Mortierella* (8%), *Fontanospora* (8%), and *Coleophoma* (3%) (data not shown), were the most widely represented in the studied cryptogamic communities.

In terms of relative abundance, *Leotiomycetes* (*Helotiales*) dominated almost all studied communities, irrespective of their nature (i.e., cover or soil below; Fig. 3A; Fig. S2). *Dothideomycetes* (*Pleosporales*) and *Mortierellomycetes* (*Mortierellales*) were among the most abundant classes in bare soils, whereas the class *Eurotiomycetes* (*Eurotiales* and *Chaetothyriales*) clearly prevailed in soils under liverwort covers and the class *Sordariomycetes* (*Sordariales*) had a remarkable abundance in soils under moss covers. In the covers, the abundance of *Agaricomycetes* (*Cantharellales*) was much higher in moss covers than in the liverwort ones.

**Fig. 4:**
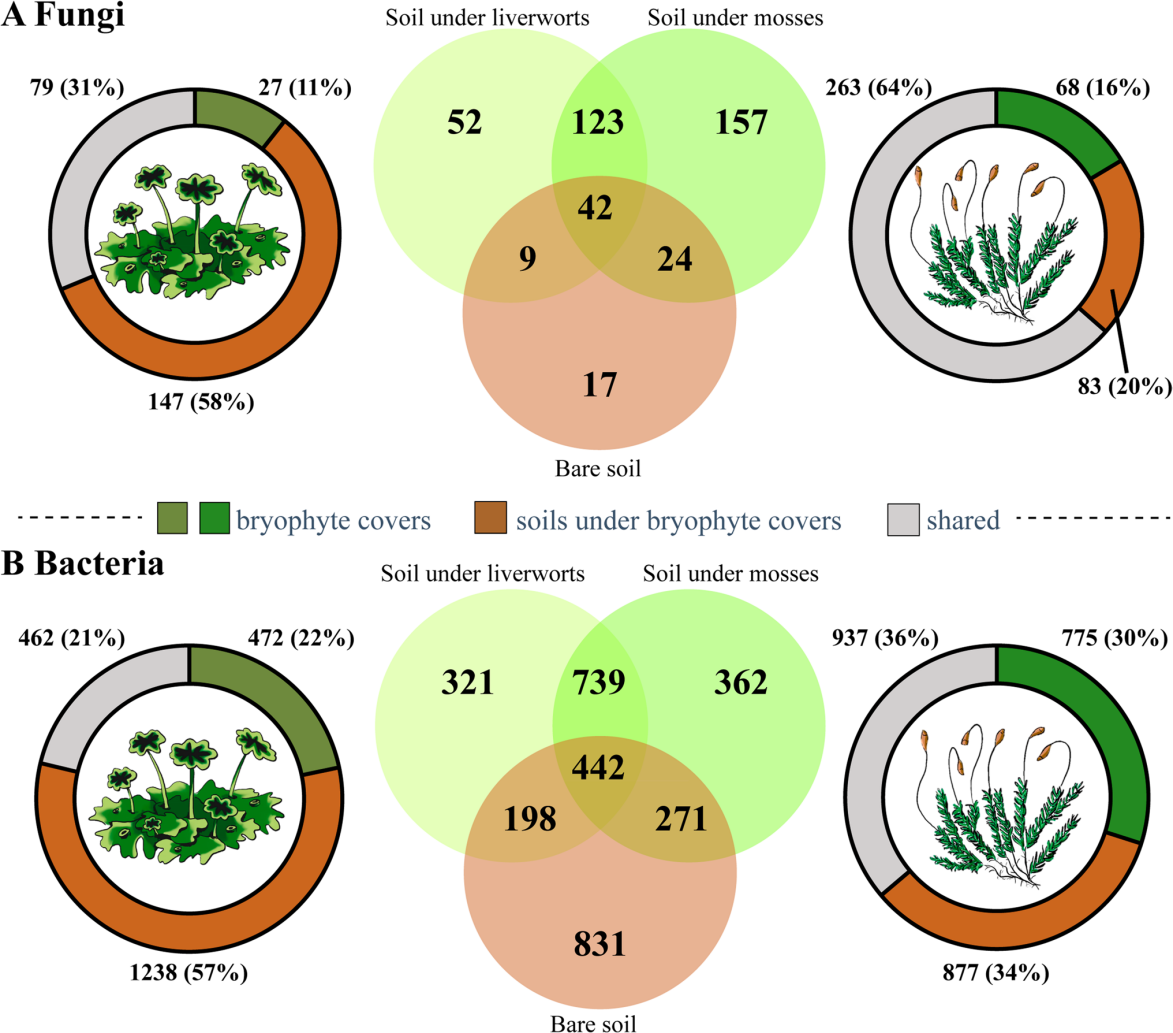
Venn diagrams of **A** fungal and **B** bacterial ASVs in different types of soil; the ring plots on the left and right of the Venn diagrams represent the number (and percentage) of ASVs shared between the bryophyte covers and soil (gray) as well as those that are exclusive to each one (greenish colors, covers; brown color, soil)

A total of 42 ASVs were shared among the three types of soil, and 123 ASVs between soils under both cryptogamic covers. Soils under mosses presented the highest number (157) of unique ASVs (Fig. 4A). While the relative proportion of unique ASVs at both covers was similar (11% liverwort and 16% moss cover), the proportion of shared ASVs between covers and underlying soil was significantly higher in moss (64%) than in liverwort (31%) covers.

### Assessment of the Bacterial Diversity, Abundance, and Specificity

**Fig. 5:**
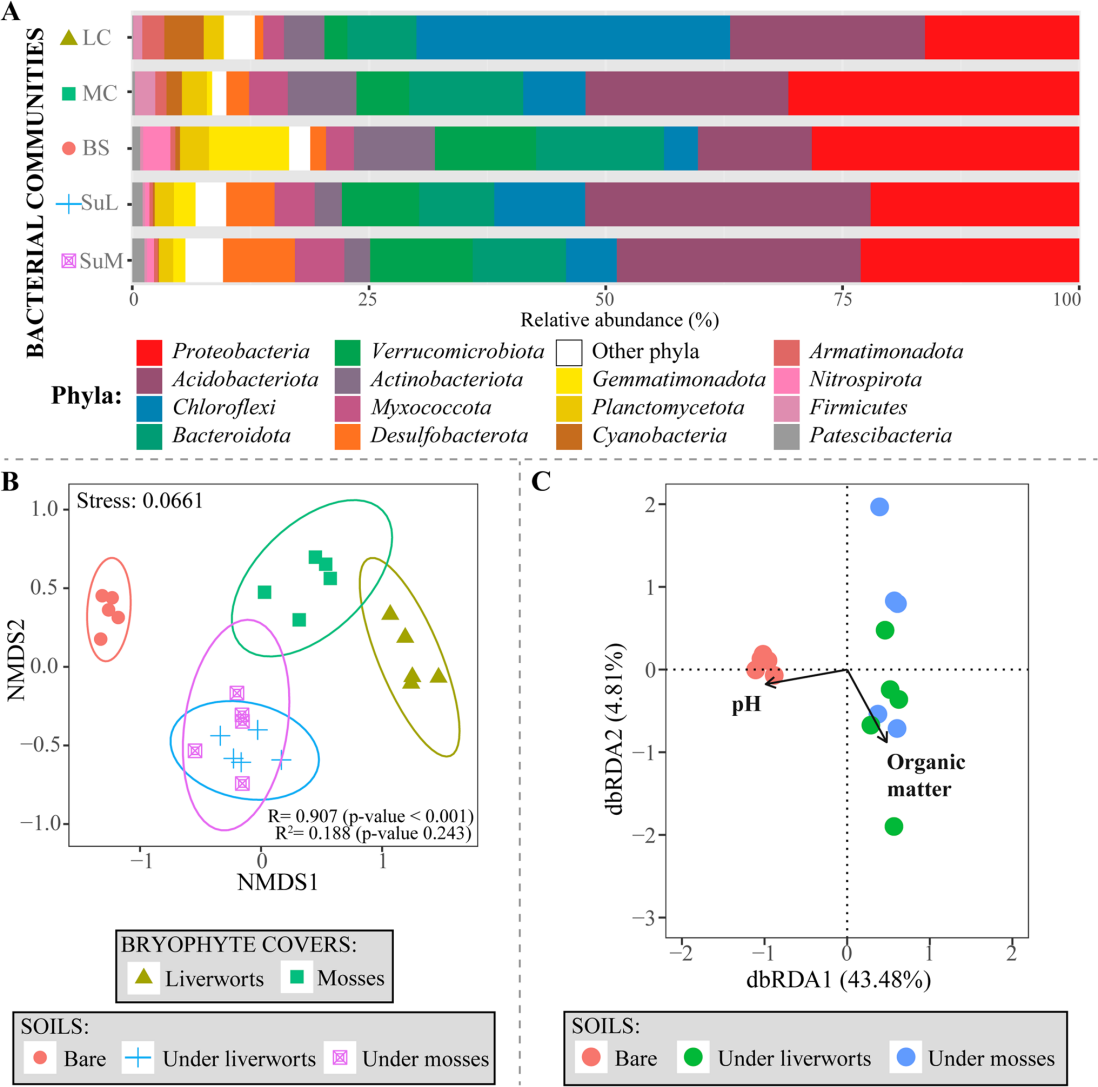
Overview of the bacterial community structure and diversity revealed by high-throughput Illumina sequencing. **A** Relative abundances at the phylum level in the five studied communities (LC, liverwort cover; MC, moss cover; BS, bare soil; SuL, soil under liverworts; SuM, soil under mosses); “other phyla” include bacterial ASVs belonging to phyla below a certain relative abundance value; **B** non-metric multidimensional scaling (NMDS) ordination plot of Bray-Curtis dissimilarities across sample categories, with ellipses representing the 95% confidence interval for a multivariate distribution and the results of ANOSIM and Adonis tests; **C** distance based redundancy analysis (db-RDA) with selected edaphic variables that explained most of the variability in the three soil bacterial communities

The number of inferred bacterial ASVs in all samples was 3766. Rarefaction curves indicated that sequencing depth was sufficient to identify the majority of bacterial ASVs in all studied communities (Fig. S1). The *Proteobacteria* (24%) and *Acidobacteriota* (22%) were in general the most abundant phyla, followed by *Chloroflexi* (12%), *Bacteroidota* (10%), and *Verrucomicrobiota* (7%) (Fig. 5A). Other phyla, such as *Gemmatimonadota*, *Cyanobacteria*, and *Firmicutes*, were found in a lower proportion. At the class level, ASVs assigned to *Acidobacteria* (16%) and *Gammaproteobacteria* (15%) were the only ones with a relative proportion above 10% (Fig. S3). *Burkholderiales* (11%), *Acidobacteriales* (8%), *Ktedonobacterales* (8%), and *Rhizobiales* (5%), and the families *Ktedonobacteraceae* (8%), *Nitrosomonadaceae* (4%), *Chitinophagaceae* (4%), *Solibacteraceae* (4%), and *Geobacteraceae* (4%) were the most representative orders and families, respectively (Table S5).

While the phyla *Proteobacteria* (*Gamma*- and *Alphaproteobacteria*) and *Acidobacteriota* (*Acidobacteria*) showed a high relative abundance in either soils or covers (Fig. 5A; Fig. S3), except for bare soils, in which *Acidobacteriota* had a lesser importance, *Bacteroidota* and *Verrucomicrobiota* showed increased abundances in either bare or bryophyte-covered soils (Fig. 5A). Remarkably, the highest relative abundance of *Chloroflexi* (*Ktedonobacteria*) was found in liverwort covers (Fig. 5A; Fig. S3). Phyla including photosynthetic species (e.g., *Cyanobacteria* or *Firmicutes*) were also more frequently found in bryophyte covers than in soils. The phylum *Gemmatimonadota* (*Gemmatimonadetes*) had a relevant contribution to the overall diversity of bare soils compared to the remaining sample categories.

A total of 442 ASVs were shared between the three soil types and the higher number of shared ASVs (739) was found between soils under both bryophyte covers, which also showed a similar number of exclusive ASVs (Fig. 4B). While a higher number of exclusive ASVs were found in the liverwort underlying soil (57%) than in the corresponding cover (22%), moss covers and the underlying soil showed a similar proportion of exclusive ASVs (30% and 34%, respectively).

### Alpha and Beta Diversities and db-RDA Analysis

**Fig. 6:**
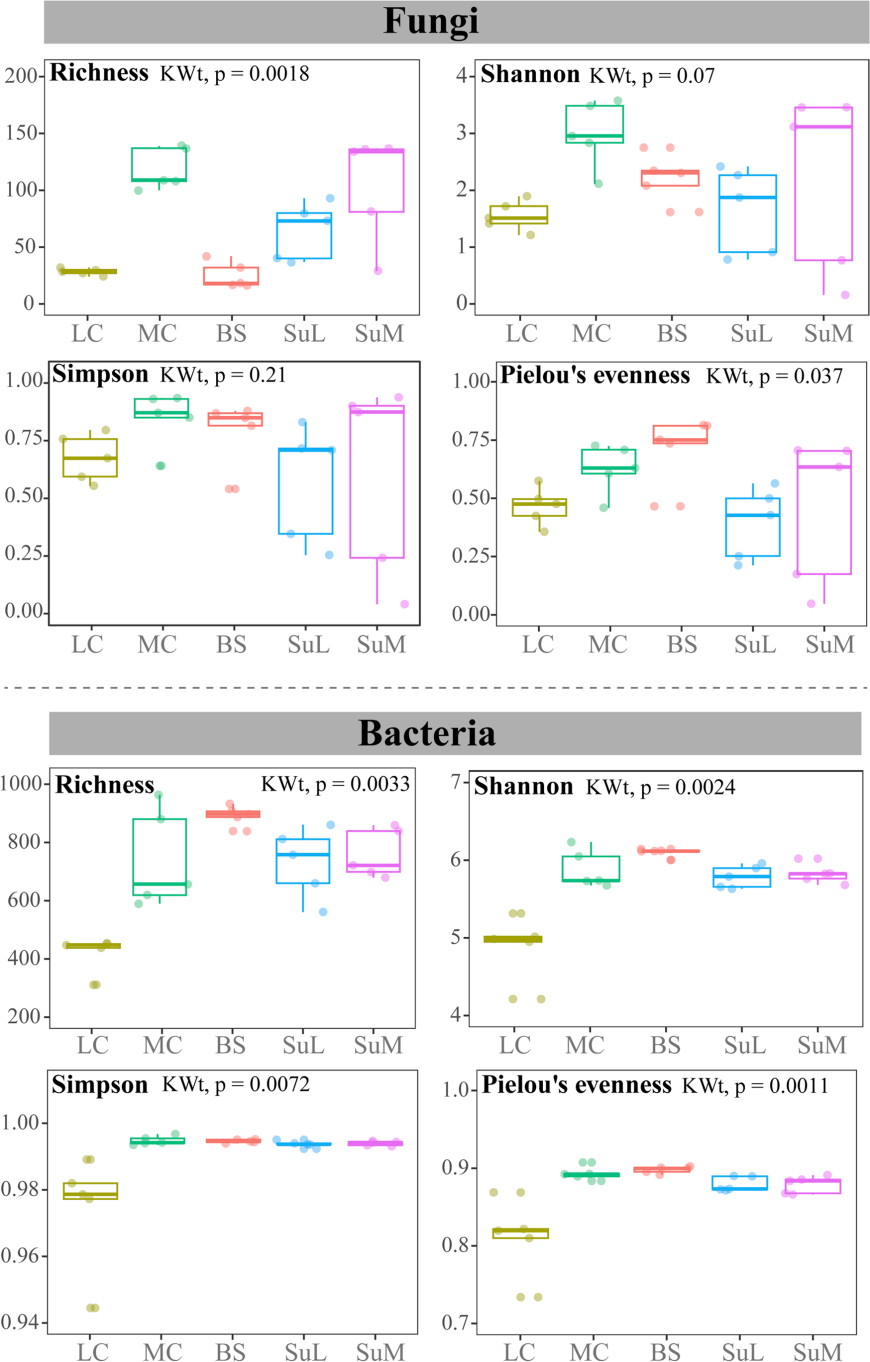
Alpha diversity estimators (richness, Shannon and Simpson index, and Pielou evenness) for fungal and bacterial communities in the different community categories (LC, liverwort cover; MC, moss cover; BS, bare soil; SuL, soil under liverworts; SuM, soil under mosses). *p* values less than 0.05 associated with the Kruskal-Wallis tests (KWt) indicate significant differences in the response variable

Alpha diversity indices for the fungal communities, including richness, Shannon, and Simpson, were higher at moss covers and their underlying soils than in the other sample categories (Fig. 6; Table S6). The lowest species richness was found in bare soils (which were also the most homogenous in the ASVs numbers according to their Pielou’s values) and soils under liverworts. Kruskal-Wallis tests indicated that differences among sample categories regarding richness and Pielou’s evenness were significant (*p* value < 0.05; Fig. 6). In bacterial communities, the highest value of alpha diversity indices was found in bare soil communities and the lowest one at liverwort covers. Differences among cryptogamic covers dominated by liverworts and the rest of the categories in all four indices were statistically supported according to Kruskal-Wallis tests (Fig. 6).

NMDS ordinations showed a remarkable segregation of fungal and bacterial communities developing in bare soils from those below moss and liverwort covers (Figs. 3B and 5B). The communities of the two bryophyte covers were separated as well. Considering fungi alone, moss cover communities and those in the underlying soil were slightly intermingled, whereas liverworts microbiome and the soil below were clearly different. In bacteria, soil communities under both bryophyte covers overlapped, but differed largely from the microbiomes of the covers themselves. Visual differences observed in the NMDS ordination diagrams were corroborated statistically with the ANOSIM test (Figs. 3B and 5B).

The db-RDA analyses were performed independently for the fungal and bacterial communities and used the soil attributes organic matter content and pH in the final model (Figs. 3C and 5C). Total variation in fungal and bacterial communities explained by dbRDA1 and dbRDA2 axes were 20.26% and 48.29%, respectively. Soil pH and organic matter content were correlated with the dbRDA1 axis and the dbRDA2 axis, respectively. Soil pH separated bare soil samples from those on which the bryophyte communities developed (Figs. 3C and 5C).

### Functional Properties of Fungal Communities

A potential ecological function was assigned to 191 fungal ASVs that could be taxonomically classified at the genus level (Fig. 3D, E; Table S7). Saprotrophy, with a relative proportion of 63.68%, was the most frequent ecological assignment. Within this nutrition mode, segregation of the different ASVs across a broad range of substrates was inferred: detritus (24.21%), soil (22.11%), wood (4.74%), dung (3.68%), or even nectar (1.05%). The second most abundant group was plant pathogenic fungi (18.42%). With lower abundances appeared root endophytes (6.84%), mycoparasites (3.68%), ectomycorrhiza (2.63%), lichenized fungi (2.11%), sooty molds (1.05%), algal parasites (0.53%), lichen parasites (0.53%), and animal parasites (0.53%).

Saprophytic soil fungi were the most abundant in bare soils, which lacked ectomycorrhizal fungi and algal, animal, and lichen parasites (Figs. 3E and S4). Saprophytic soil and detritus fungi were the most abundant in liverworts and mosses covers, respectively. Lichenized, nectar saprophytes, and lichen parasitic fungi were exclusive of soil edaphic communities. On the other hand, animal and algal parasites were only found in the studied cryptogamic covers (Figs. 3E and S4).

### Cryptogamic Cover Structure

**Fig. 7:**
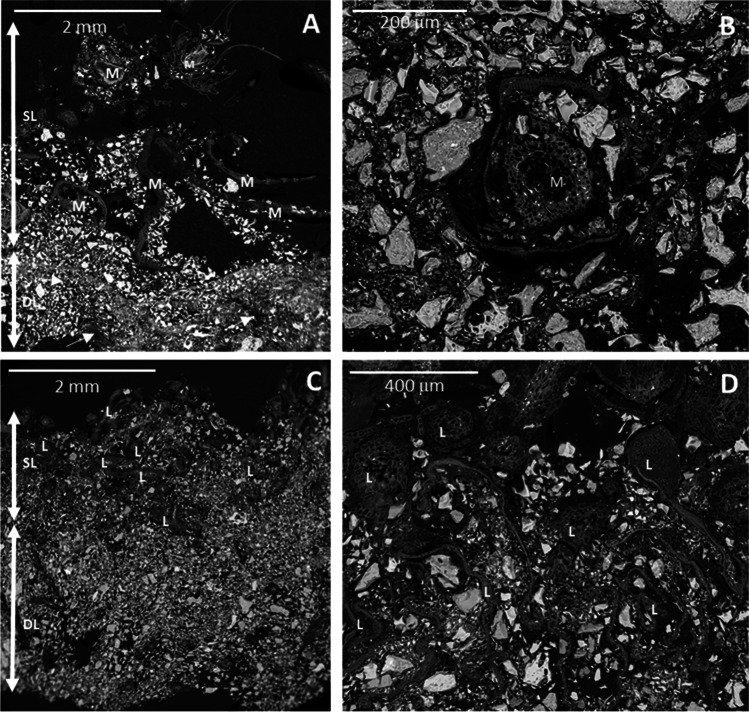
Images of cross sections of cryptogamic covers dominated by mosses (**A**–**B**) and liverworts (**C**–**D**) with backscattered electrons in a scanning electron microscope. **A** and **C** show the superficial layer (SL) and the deeper layer (DL) in both types of cryptogamic cover. **B** and **D** correspond to the superficial layer of cryptogamic covers dominated by mosses (**B**) and liverworts (**D**). M (moss), L (liverwort), and arrows note moss remnants in the deeper layer of a moss-dominated cover

Moss-dominated cryptogamic covers were structurally heterogeneous, with a superficial layer (SL) formed by moss living structures and non-aggregated soil particles (Fig. 7A), and a deeper (DL), more compact layer with moss remnants embedded in a dense mineral matrix (arrows in Fig. 7A, B). Liverwort-dominated cryptogamic covers were also composite structures with thalli concentrated in a superficial layer (SL) and a deeper layer (DL) consisting of mainly aggregated mineral fragments (Fig. 7C, D). However, liverwort thalli of the superficial layer were totally immersed in a dense mineral matrix (Fig. 7D) resulting in a more compact superficial layer with higher mineral content than the superficial layer of moss-dominated cryptogamic covers. Hence, liverwort-dominated cryptogamic covers showed less differentiation between superficial and deeper layer and consequently were structurally more homogenous.

## Discussion

The present study demonstrates that the development of bryophyte cryptogamic covers in the Icelandic Highlands produces an extraordinary change in microbial community diversity and composition as compared with soils devoid of such covers. Covered and bare soils also differ in their abiotic attributes, a finding that highlights the relationships between microbial community composition and soil biogeochemistry. Furthermore, moss and liverwort covers harbor fungal and bacterial communities that differ in their structure, and these differences are also observed in the underlying soils, particularly for fungi.

The ascomycete fungal class *Leotiomycetes* showed the highest overall relative abundance in the studied bryophyte covers, a finding that may align with the tight ecological link between this fungal lineage and plants, as it encompasses numerous pathogenic, endophyte, saprophyte, or symbiont species [60, 61]. Specifically, ASVs assigned to the genera *Coleophoma*, *Botrytis*, *Hyaloscypha*, or *Pezoloma*, which host bryophyte parasitic and saprophytic species, were frequent in the assembled dataset [11, 62–64]. Although diverse symbiotic associations between fungi and mosses have been previously described [65], there are virtually no data on symbiotic associations between fungi and liverworts of the family *Antheliaceae*, to which the species dominating the study area belongs [66]. For this reason, the fungi that are found in liverwort-dominated covers might be either specific or opportunistic saprophytes, although it is also possible that their presence in the thalli is due to stochastic factors (i.e., transient species). For example, lichens have been shown to host a relevant proportion of transient fungal species developing on their thalli [67]. On the other hand, *Eurotiomycetes* was the most abundant fungal class in the soils underlying these bryophyte covers, its proportion being noticeably high under liverworts. This finding may be explained by the abundance of sequences assigned to the orders *Eurotiales* and *Chaetothyriales*, which include many plant saprophytic fungi [60, 68]. This high abundance of *Chaetothyriales* is consistent with that observed in other studies on *Anthelia*-dominated covers [35]. However, the relative abundance of *Eurotiomycetes* was almost negligible in bare soils. Taxa assigned to the classes *Dothideomycetes* and *Mortierellomycetes* predominated in these soils, which include a high number of soil saprophytic species [60, 69]. *Eurotiomycetes* and *Dothideomycetes* constitute the so-called black fungi, because of their dark, melanin-based pigmentation, and partly due to this characteristic, these fungi are resistant to multiple types of stress, ranging from UV radiation to heat and desiccation [70]. Their predominance in the studied Icelandic soils and cryptogamic covers might be linked to the relatively extreme abiotic conditions that characterize this region.

The results obtained in the present study indicate that there is a certain specificity of fungal taxa by type of cryptogamic cover according to the dominant bryophyte. For example, moss covers showed a greater abundance of basidiomycetes (*Agaricales*, *Agaricomycetes*), which might be involved in mutualistic relationships (e.g., mycorrhizae) [71]. Species of the vascular plant genera *Salix* and *Betula* co-occurred with mosses in the study area, and these plants often form ectomycorrhizal associations with agaricomycete species of the genera *Cortinarius* and *Russula* [72, 73]. Recently, members of these fungal genera were described in association with mosses [74]. Interestingly, ASVs assigned to the fungus *Lecophagus* (*Orbiliomycetes*) were found in moss covers; several species of this genus, like *L. muscicola* and *L. antarcticus*, the latter described in maritime Antarctica, are carnivorous, trapping rotifers and tardigrades using specialized hyphae structures [75].

Regarding the composition of bacterial communities, *Proteobacteria* and *Acidobacteriota* were the most abundant, both in soils, including those that lacked a bryophyte cover, as well as in covers, a result that aligns with previous findings in similar habitats in other geographic areas [57, 76]. In fact, members of these phyla belong to the “core microbiome” of Arctic soils [30], that is, they show a wide distribution and, probably, ecological non-specificity in this region. *Proteobacteria* include photoautotrophic and chemolithotrophic organisms, some of which have the ability to fix nitrogen and establish symbiotic relationships with bryophytes [76], which could be key to the functioning of the studied Icelandic cryptogamic covers. *Acidobacteriota* are also frequent in edaphic environments and present a metabolism adapted to oligotrophy [77] or adverse environmental conditions, such as those existing in the study area, especially during winters. The *Verrucomicrobiota* phylum, which includes methanotrophic bacteria, was abundant in soils under cryptogamic covers. These bacteria are frequent in soils with oligotrophic and anoxic conditions [78], such as those that could be generated in the study area in summer by waterlogging after ice melting.

The *Bacteroidota* and *Actinobacteriota* showed a moderate abundance in moss-dominated covers. Members of both phyla have been previously described in association with moss phyllidia and could provide some protection to mosses against freezing [79]. Contrary to moss covers, liverwort covers hosted a significantly great proportion of *Chloroflexi* (*Ktedonobacteria*). Members of this bacterial phylum are credited with the ability to degrade organic matter, so its presence in these samples could be associated with the decomposition of liverwort fragments embedded in the mineral matrix of the cover [64, 80]. Opposite to the present work, previous studies that analyzed the microbiome of the first 5 mm in biological crusts of *Anthelia* did not find *Chloroflexi* to be the most abundant phylum [25]. These apparently contradicting findings suggest that the distribution of microorganisms is not homogeneous throughout the depth of the cover and that *Chloroflexi* could be more abundant at greater depths where bryophyte remnants are probably more degraded. In addition, photosynthetic bacteria such as *Cyanobacteria* were common in both covers, but almost absent in the soils, similar to what occurs in deglaciated soils of Tierra del Fuego [10, 81]. This association may promote the input of N and nutrients into the covers [81]. Other primary producers could be *Chloroflexi* and *Firmicutes* [82], which were also frequently detected in covers of liverworts and mosses, respectively. Indeed, our analyses of soil attributes showed higher C and N contents in soils under both bryophytes compared to bare soils.

Differences in microbial communities among different types of covers (e.g., mosses vs lichens) were previously reported [16, 83–85]. The present study provides further evidence for changes in composition and structure of edaphic microbial communities after the establishment of both bryophyte-dominated cryptogamic covers [86–88]. Cryptogamic covers dominated by different organisms (e.g., cyanobacteria, bryophytes, or lichens) along different succession stages impose changes in the soil microbiome. While early successional stages are dominated by microorganisms with the ability to fix carbon and nitrogen, thus accumulating the nutrients of the upper layer of the soil, covers in latter successional stages are dominated by microorganisms with the ability to degrade complex compounds, providing nutrients for the establishment of vascular plants [89]. Although the development of moss and liverwort covers has been shown in the present study to induce similar effects on soil biogeochemical properties (soil attributes were similar under both types of bryophytes covers and these differed markedly from bare soils), the dominant bryophyte seems to have specific influence on community structure in the soils below them, because the relative abundance of certain microbial taxa differed. To our knowledge, this is the first time that differences between the microbiome of cryptogamic covers dominated by mosses and liverworts in the same area are reported. These differences could be related to structural or/and anatomical differences of the cover itself [90]. While the structure of liverworts covers was more compact and their thalli appeared totally embedded in a dense mineral matrix [91], cryptogamic covers dominated by mosses were more structurally heterogeneous. This structural heterogeneity of moss covers might favor the formation of additional microhabitats which favor the establishment of more diverse microbial communities [90]. On the other hand, the differences in mineral content at the superficial layer of both types of bryophytes-dominated covers could also have influence on the microbiome composition because the mineral-microorganism interactions are regarded to be quite specific [92, 93].

In future climate scenarios, the development of cryptogamic covers in tundra ecosystems will be predictably favored [94, 95]. The relative ability of different aboveground cryptogams to thrive under the new conditions might induce a great effect on the edaphic microbial community composition and diversity, and consequently on the responses of polar ecosystems to environmental changes. In addition, the ability of polar soils to exchange atmospheric greenhouse gases has been recently attributed to differential effects of dominant cryptogams and, therefore, on the activity of below-ground microbial communities [96]. Hence, our findings are essential to understand and predict the biotic responses of polar ecosystems to future climate change.

## Conclusions

This study demonstrated that the establishment of bryophyte-dominated cryptogamic covers (i) is associated to differences in the abiotic soil attributes and (ii) influences the composition and structure of bacterial and fungal communities in the underlying soils. In addition, our findings prove that the response of edaphic microbial communities to the establishment of cryptogamic covers is controlled by the dominating bryophyte, which may be linked to fine-tuned interactions between certain microorganisms and specific bryophytes, as well as to structural differences between both bryophyte cover types. Different sensitivity to climate change of both cryptogams could have a great influence on polar edaphic communities.

## Supplementary information

ESM 1 (PDF 528 kb)

## Data Availability

The data sets generated for this study can be found in the NCBI Sequence Read Archive the with BioProject number PRJNA917534.
