# Supplementary material for: Moss and Liverwort Covers Structure Soil Bacterial and Fungal Communities Differently in the Icelandic Highlands
Source: Microb Ecol. 2023 Feb 18;86(3):1893–908. doi: 10.1007/s00248-023-02194-x (PMC10497656; doi:10.1007/s00248-023-02194-x)
Supplement: Supplementary file 1 — (PDF 528 kb) [file 248_2023_2194_MOESM1_ESM.pdf]

**SUPPLEMENTARY MATERIAL for article:**

# Moss and liverwort covers structure soil bacterial and fungal communities differently in the Icelandic Highlands

Javier Ortiz-Rivero[1], Isaac Garrido-Benavent[2], Starri Heiðmarsson[3, 4] & Asunción de los Ríos[1]

[1] Department of Biogeochemistry and Microbial Ecology, National Museum of Natural Sciences (MNCN-CSIC), C/ Serrano 115 dpdo, E-28045 Madrid, Spain. E-mail: javierortiz.96@hotmail.com; ORCID: 0000-0002-2978-1694. E-mail: arios@mncn.csic.es. ORCID: 0000-0002-0266-3516.

[2] Departament de Botànica i Geologia, Fac. CC. Biològiques, Universitat de València, C/ Doctor Moliner 50, E-46100 Burjassot, Valencia, Spain. E-mail: Isaac.Garrido@uv.es; ORCID: 0000-0002-5230-225X

[3] Icelandic Institute of Natural History, Akureyri Division, Borgir Nordurslod, 600 Akureyri, Iceland

[4] Present address: Northwest Iceland Nature Research Centre, Aðalgötu 2, 550 Sauðárkrókur, Iceland. E-mail: starri@nnv.is ORCID: 0000-0003-0329-6882

Author for correspondence: Isaac Garrido-Benavent

# Rarefaction curves-Fungi

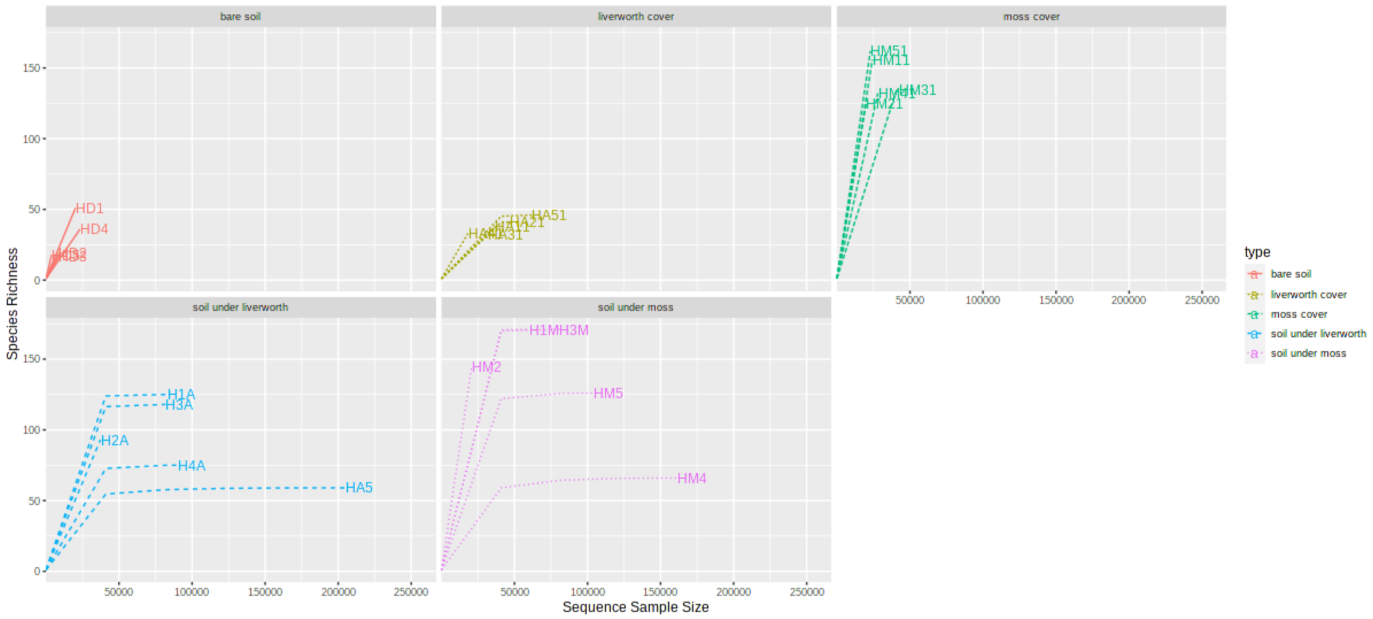


# Rarefaction curves-Bacteria

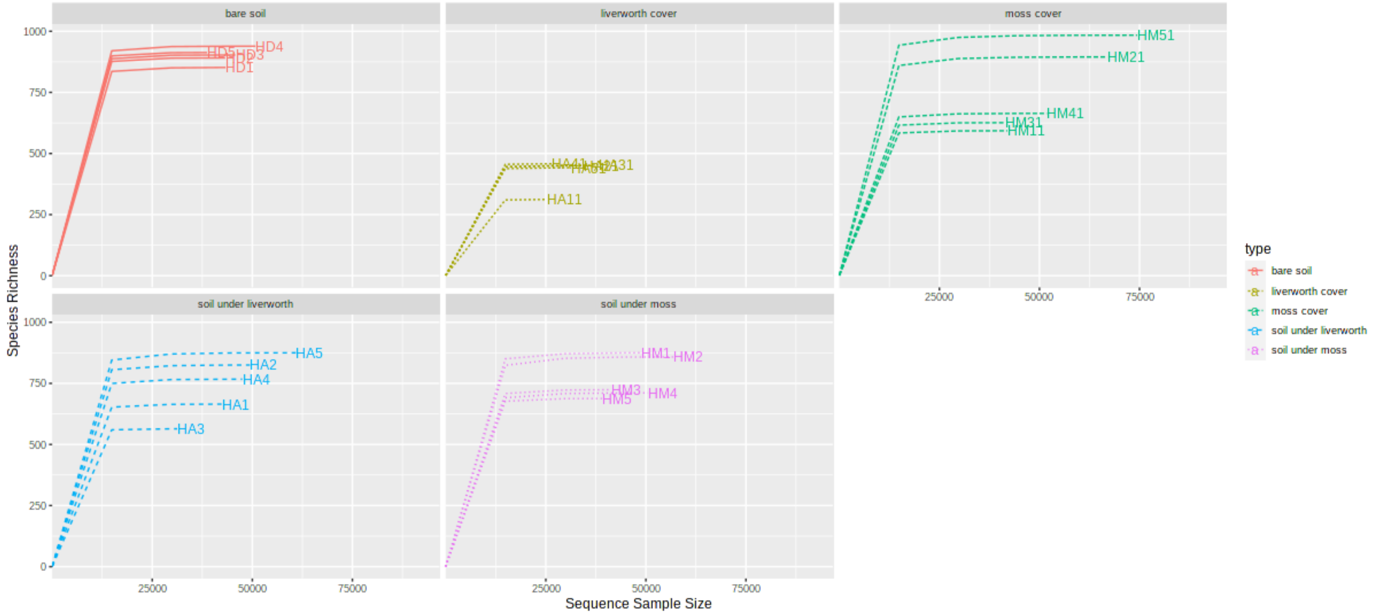


**Fig. S1** Rarefaction curves of the fungal and bacterial communities.

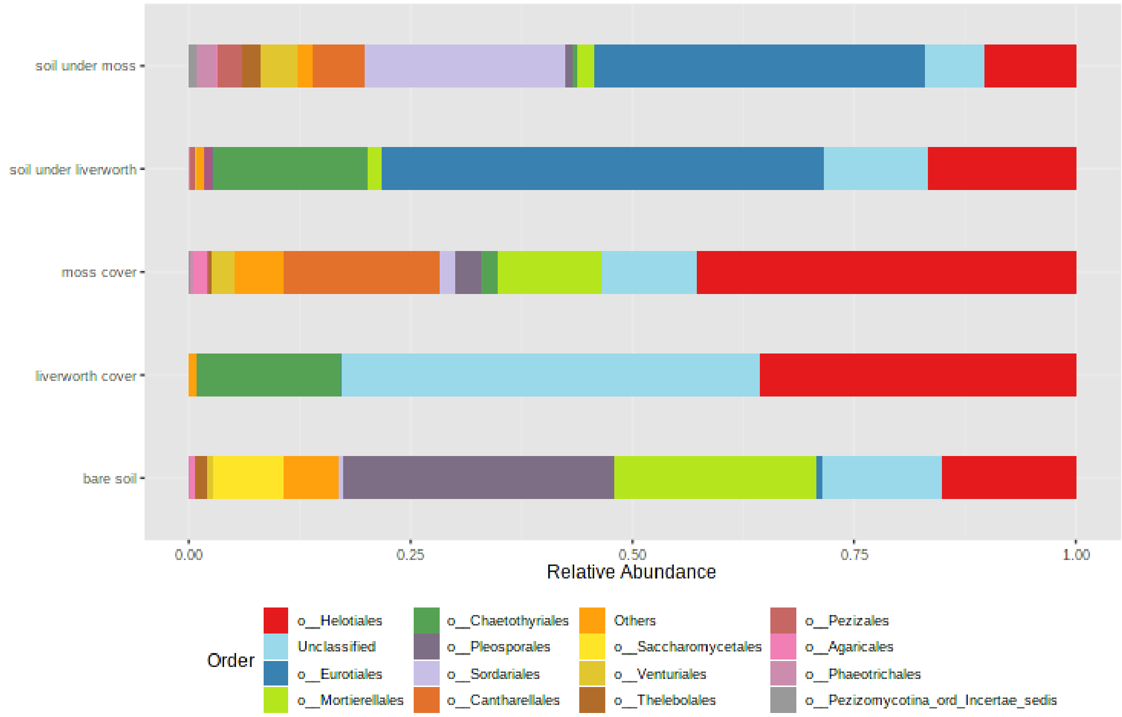


**Fig. S2** Relative abundances of fungal communities at the order level identified by high-throughput Illumina sequencing. The 15 most abundant orders are represented in the different studied categories.

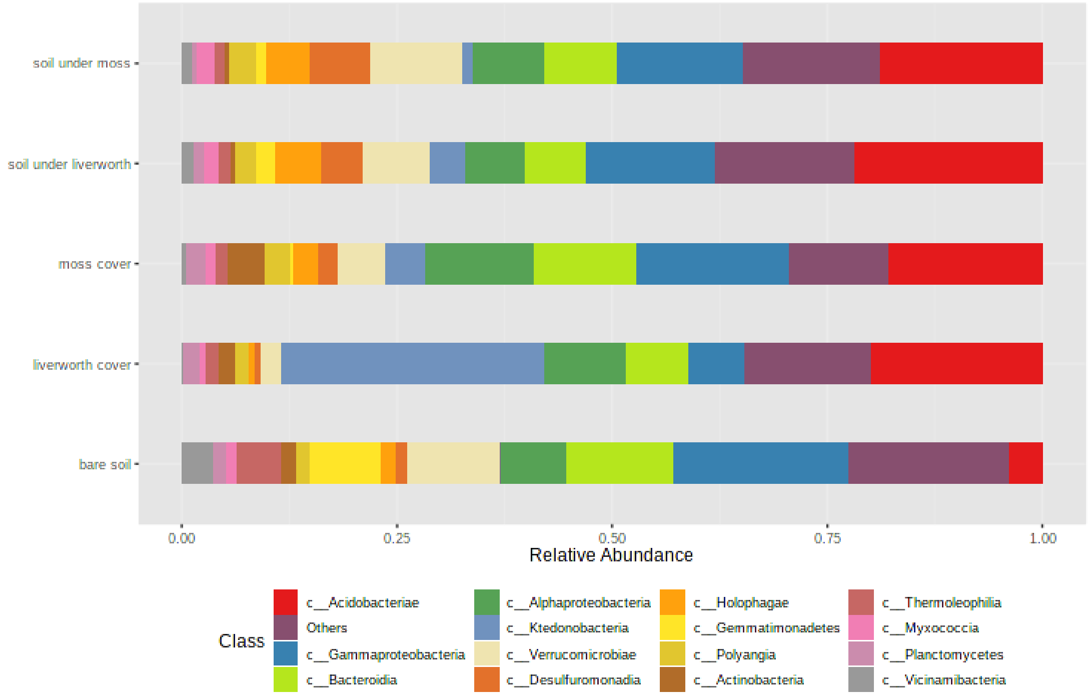


**Fig. S3** Relative abundances of bacterial communities at the class level identified by high-throughput Illumina sequencing. The 15 most abundant bacterial classes are represented in the different studied categories.

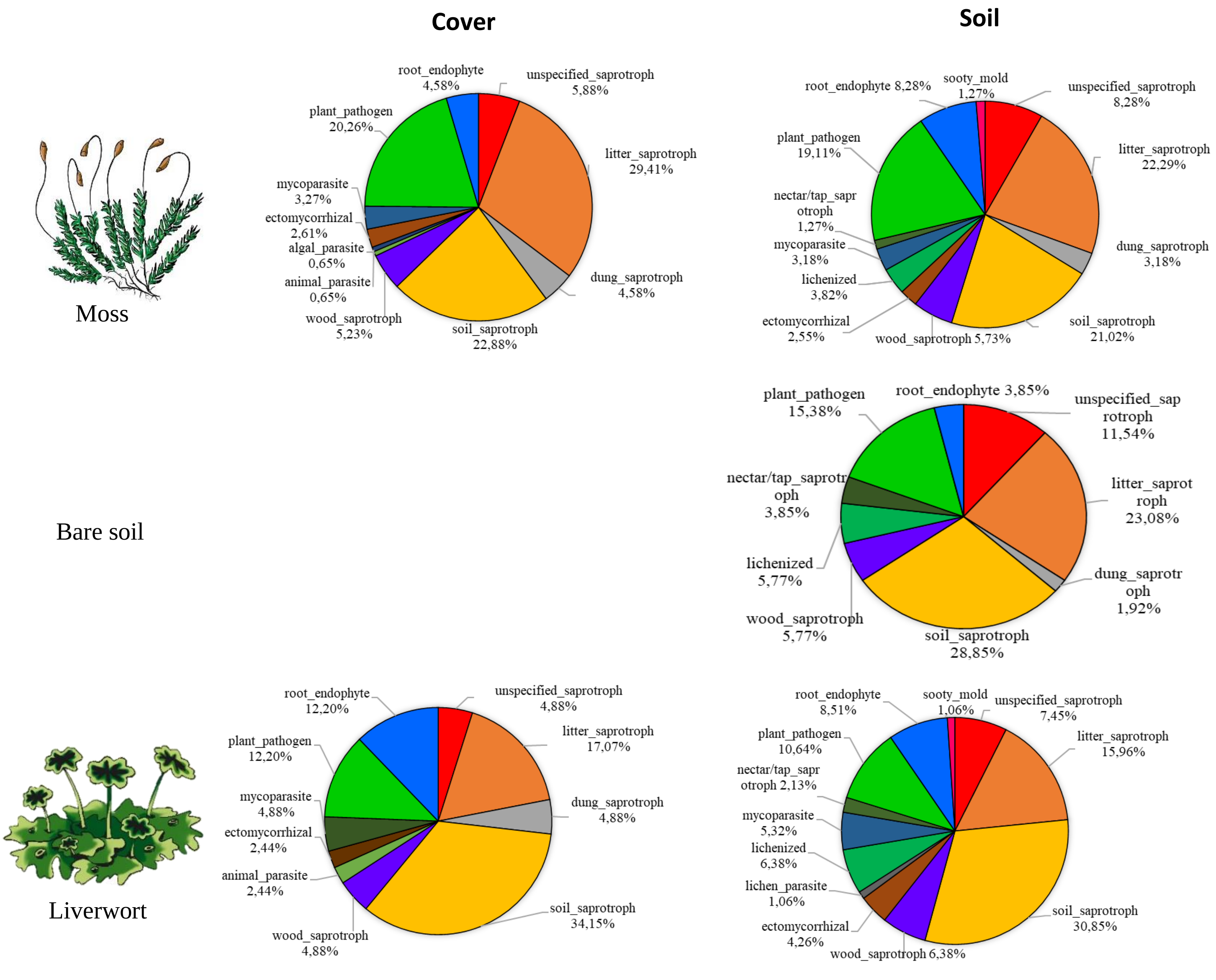


**Fig. S4** Ecological role of fungal ASVs at the genus level in each of the categories (soils and covers).

**Table S1.** Soil physicochemical properties in the different types of soils: bare soil (HD1-HD5), soils under liverworts (HA1-HA5), soils under moss (HM1-HM5) and in the cryptogamic covers: liverwort cover (HA11-HA51) and moss cover (HM11-HM51)

| Soil | N g/100g | C g/100g | C/N | pH | M.O % |
|---|---|---|---|---|---|
| HD1 | 0,02 | 0,09 | 3,87 | 6,68 | 1,82 |
| HD2 | 0,03 | 0,10 | 3,80 | 7,17 | 1,84 |
| HD3 | 0,01 | 0,04 | 3,05 | 7,09 | 1,83 |
| HD4 | 0,03 | 0,07 | 2,54 | 7,03 | 1,41 |
| HD5 | 0,01 | 0,05 | 5,68 | 6,98 | 1,68 |
| HA1 | 0,09 | 1,23 | 13,93 | 6,17 | 3,64 |
| HA2 | 0,08 | 1,64 | 19,50 | 5,93 | 3,72 |
| HA3 | 0,09 | 1,83 | 21,28 | 6,00 | 6,19 |
| HA4 | 0,07 | 1,34 | 19,03 | 6,16 | 24,53 |
| HA5 | 0,11 | 2,73 | 25,63 | 6,46 | 4,49 |
| HM1 | 0,08 | 1,24 | 15,24 | 5,87 | 4,29 |
| HM2 | 0,05 | 0,90 | 17,80 | 6,13 | 3,21 |
| HM3 | 0,06 | 0,67 | 11,85 | 5,57 | 2,27 |
| HM4 | 0,11 | 3,04 | 28,03 | 5,67 | 8,50 |
| HM5 | 0,11 | 2,01 | 18,49 | 6,00 | 5,67 |

| Cover | N g/100g | C g/100g | C/N |
|---|---|---|---|
| HA11 | 0,12 | 4,14 | 34,10 |
| HA21 | 0,20 | 6,75 | 33,97 |
| HA31 | 0,21 | 8,30 | 40,00 |
| HA41 | 0,28 | 7,91 | 28,63 |
| HA51 | 0,24 | 8,27 | 34,13 |
| HM11 | 0,08 | 2,01 | 24,41 |
| HM21 | 0,09 | 1,94 | 22,51 |
| HM31 | 0,08 | 1,52 | 18,84 |
| HM41 | 0,05 | 0,72 | 15,50 |
| HM51 | 0,09 | 2,71 | 29,85 |

**Table S2.** ANOVA/Kruskal-Wallis tests results of the soil attributes analyzed

| Soil attributes | Statistical test | Normality assumption | Heteroscedasticity assumption | p-value |
|---|---|---|---|---|
| Nitrogen | Kruskal-Wallis | NO | YES | 0,0009222 |
| Carbon | Kruskal-Wallis | NO | YES | 0,0009346 |
| Carbon/Nitrogen | ANOVA | YES | YES | 0,0001165 |
| Organic matter | Kruskal-Wallis | NO | YES | 0,008148 |
| pH | ANOVA | YES | YES | 4,58e-06 |

**Table S3.** Summary of the fungal classes found in the set of all samples

| Phylum | *Ascomycota* | *Basidiomycota* | *Mortierellomycota* | *Rozellomycota* |
|---|---|---|---|---|
| Class | *Dothideomycetes*<br>*Eurotiomycetes*<br>*Lecanoromycetes*<br>*Leotiomycetes*<br>*Orbiliomycetes*<br>*Pezizomycetes*<br>*Pezizomycotina*<br>*Saccharomycetes*<br>*Sordariomycetes* | *Agaricomycetes*<br>*Cystobasidiomycetes*<br>*Microbotryomycetes*<br>*Pucciniomycetes*<br>*Tremellomycetes* | *Mortierellomycetes* | *Rozellomycetes* |

**Table S4.** Summary of the fungal orders found in the set of all samples

| Phylum | *Ascomycota* | *Basidiomycota* | *Mortierellomycota* | *Rozellomycota* |
|---|---|---|---|---|
| **Order** | *Caliciales*<br>*Capnodiales*<br>*Chaetothyriales*<br>*Dothideales*<br>*Eurotiales*<br>*Helotiales*<br>*Hypocreales*<br>*Magnaporthales*<br>*Orbiliales*<br>*Petilgerales*<br>*Pezizales*<br>*Pezizomycotina*<br>*Phaeotrichales*<br>*Pleosporales*<br>*Saccharomycetales*<br>*Sordariales*<br>*Teloschistales*<br>*Thelebolales*<br>*Venturiales*<br>*Verrucariales*<br>*Xylariales* | *Agaricales*<br>*Atheliales*<br>*Cantharellales*<br>*Cystofilobasidiales*<br>*Filobasidiales*<br>*Kriegeriales*<br>*Platygloeales*<br>*Sebacinales*<br>*Sporidiobolales*<br>*Thelephorales*<br>*Tremellales* | *Mortierellales* | *Rozellales* |

**Table S5.** Taxonomic information down to family level in the set of all samples of bacterial ASVs

| Phylum | Class | Order | Family |
|---|---|---|---|
| *Acidobacteriota* | *Acidimicrobiia* | *Acetobacterales* | *Acetobacteraceae* |
| *Actinobacteriota* | *Acidobacteriae* | *Acidobacteriales* | *Acidobacteriaceae_* |
| *Armatimonadota* | *Actinobacteria* | *Bacteroidales* | *Anaeromyxobacteraceae* |
| *Bacteroidota* | *Alphaproteobacteria* | *Bryobacterales* | *Bryobacteraceae* |
| *Bdellovibrionota* | *Anaerolineae* | *Burkholderiales* | *Chitinophagaceae* |
| *Chloroflexi* | *Bacteroidia* | *Chitinophagales* | *Chthoniobacteraceae* |
| *Cyanobacteria* | *Blastocatellia* | *Chthoniobacterales* | *Comamonadaceae* |
| *Dependentiae* | *Cyanobacteriia* | *Cytophagales* | *Gemmatimonadaceae* |
| *Desulfobacterota* | *Desulfuromonadia* | *Gaiellales* | *Geobacteraceae* |
| *Elusimicrobiota* | *Gammaproteobacteria* | *Gemmatimonadales* | *Holophagaceae* |
| *Fibrobacterota* | *Gemmatimonadetes* | *Geobacterales* | KD4_96 |
| *Firmicutes* | *Holophagae* | KD4_96 | *Ktedonobacteraceae* |
| *Gemmatimonadota* | KD4_96 | *Ktedonobacterales* | *Nitrosomonadaceae* |
| *Latescibacterota* | *Ktedonobacteria* | *Myxococcales* | *Opitutaceae* |
| MBNT15 | Lineage_IIa | *Opitutales* | *Pedosphaeraceae* |
| *Methylomirabilota* | *Myxococcia* | *Pedosphaerales* | *Rhizobiales_Incertae_Sedis* |
| *Myxococcota* | *Nitrospiria* | *Polyangiales* | *Solibacteraceae* |
| Nitrospirota | Parcubacteria | Rhizobiales | Sphingobacteriaceae |
| *Patescibacteria* | *Planctomycetes* | *Solibacterales* | Subgroup_2 |
| *Planctomycetota* | *Polyangia* | *Sphingobacteriales* | Subgroup_7 |
| *Proteobacteria* | *Thermoleophilia* | Subgroup_2 | uncultured |

| | | | |
|---|---|---|---|
| *Spirochaetota* | *Verrucomicrobiae* | Subgroup_7 | WD260 |
| *Verrucomicrobiota* | *Vicinamibacteria* | *Vicinamibacterales* | *Xanthobacteraceae* |
| WPS_2 | WPS_2 | WD260 | |

**Table S6.** Average values of the alpha-diversity index (Fungi/Bacteria) in each of the categories

| Community | Richness | Shannon index | Simpson index | Pielou's evenness |
|---|---|---|---|---|
| Bare soil | 25/892,4 | 2,22/6,1 | 0,79/0,9946 | 0,72/0,898 |
| Soil under moss | 103,4/759,4 | 2,19/5,825 | 0,6/0,994 | 0,45/0,879 |
| Soil under liverwort | 64,6/730,2 | 1,65/5,788 | 0,57/0,9937 | 0,39/0,879 |
| Moss cover | 118,6/741,8 | 2,99/5,886 | 0,85/0,9948 | 0,63/0,893 |
| Liverwort cover | 28,2/420,4 | 1,55/4,896 | 0,68/0,9743 | 0,47/0,811 |

**Table S7.** Main ecological function of those fungal ASVs with taxonomic assignment at the genus level, using the information contained in the FungalTraits database [58].

| Genus | Primary lifestyle | Genus | Primary lifestyle |
|---|---|---|---|
| *Alternaria* | Plant pathogen | *Mycocentrospora* | Plant pathogen |
| *Arrhenia* | Litter saprotroph | *Mycosymbioces* | Mycoparasite |
| *Aspergillus* | Unspecified saprotroph | *Naganishia* | Unspecified saprotroph |
| *Aureobasidium* | Sooty mold | *Neobulgaria* | Wood saprotroph |
| *Bannozyma* | Soil saprotroph | *Obtectodiscus* | Litter saprotroph |
| *Botrytis* | Plant pathogen | *Ophiosphaerella* | Plant pathogen |
| *Bryoglossum* | Litter saprotroph | *Peltigera* | Lichenized |
| *Buellia* | Lichenized | *Penicillium* | Unspecified saprotroph |
| *Cadophora* | Litter saprotroph | *Pezizella* | Root endophyte |
| *Cladophialophora* | Soil saprotroph | *Pezoloma* | Root endophyte |
| *Clathrosphaerina* | Litter saprotroph | *Phaeosphaeria* | Litter saprotroph |
| *Coleophoma* | Plant pathogen | *Phaeotremella* | Mycoparasite |
| *Cortinarius* | Ectomycorrhizal | *Phenoliferia* | Unspecified saprotroph |
| *Cystobasidium* | Mycoparasite | *Phialocephala* | Soil saprotroph |
| *Deconica* | Litter saprotroph | *Podospora* | Dung saprotroph |
| *Dekkera* | Nectar/tap saprotroph | *Preussia* | Dung saprotroph |
| *Dioszegia* | Litter saprotroph | *Ramularia* | Plant pathogen |
| *Endophoma* | Soil saprotroph | *Rhodotorula* | Unspecified saprotroph |
| *Fontanospora* | Litter saprotroph | *Rimbachia* | Plant pathogen |
| *Fusicladium* | Plant pathogen | *Russula* | Ectomycorrhizal |
| *Gaeumannomyces* | Plant pathogen | *Sanchytrium* | Algal parasite |
| *Glarea* | Soil saprotroph | *Schizothecium* | Dung saprotroph |
| *Gloeotinia* | Plant pathogen | *Scutellinia* | Wood saprotroph |
| *Goffeauzyma* | Soil saprotroph | *Serendipita* | Root endophyte |
| *Gorgomyces* | Litter saprotroph | *Sistotrema* | Litter saprotroph |
| *Gyoerffyella* | Litter saprotroph | *Solicoccozyma* | Soil saprotroph |
| *Hyalopeziza* | Litter saprotroph | *Spirosphaera* | Litter saprotroph |
| *Hyaloscypha* | Litter saprotroph | *Sporormiella* | Litter saprotroph |
| *Isthmolongispora* | Litter saprotroph | *Taeniospora* | Unspecified saprotroph |
| *Itersonilia* | Plant pathogen | *Tetracladium* | Litter saprotroph |
| *Kriegeria* | Plant pathogen | *Tomentella* | Ectomycorrhizal |
| *Lachnellula* | Wood saprotroph | *Tremella* | Mycoparasite |
| *Lachnum* | Litter saprotroph | *Trichocladium* | Unspecified saprotroph |
| *Laetinaevia* | Litter saprotroph | *Trichodelitschia* | Dung saprotroph |
| *Lasiobelonium* | Wood saprotroph | *Trichoderma* | Mycoparasite |
| *Lecophagus* | Animal parasite | *Truncatella* | Plant pathogen |
| *Lecythophora* | Unspecified saprotroph | *Ustilentyloma* | Plant pathogen |
| *Microdochium* | Plant pathogen | *Varicosporium* | Litter saprotroph |
| *Monodictys* | Lichen parasite | *Venturia* | Plant pathogen |
| *Mortierella* | Soil saprotroph | *Verrucaria* | Lichenized |
| *Mrakia* | Unspecified saprotroph | *Vishniacozyma* | Soil saprotroph |
